# The Role of Gut Microbiota and Metabolites in Obesity-Associated Chronic Gastrointestinal Disorders

**DOI:** 10.3390/nu14030624

**Published:** 2022-01-31

**Authors:** Maafi R. Islam, Subha Arthur, Jennifer Haynes, Molly R. Butts, Niraj Nepal, Uma Sundaram

**Affiliations:** Department of Clinical and Translational Sciences, Joan C. Edwards School of Medicine, Marshall University, Huntington, WV 25701, USA; islam8@marshall.edu (M.R.I.); arthursu@marshall.edu (S.A.); haynesje@marshall.edu (J.H.); buttsmolly@gmail.com (M.R.B.); nepal@marshall.edu (N.N.)

**Keywords:** gut microbiota, obesity, IBD, CRC, SCFAs

## Abstract

The gut microbiota is a complex community of microorganisms that has become a new focus of attention due to its association with numerous human diseases. Research over the last few decades has shown that the gut microbiota plays a considerable role in regulating intestinal homeostasis, and disruption to the microbial community has been linked to chronic disease conditions such as inflammatory bowel disease (IBD), colorectal cancer (CRC), and obesity. Obesity has become a global pandemic, and its prevalence is increasing worldwide mostly in Western countries due to a sedentary lifestyle and consumption of high-fat/high-sugar diets. Obesity-mediated gut microbiota alterations have been associated with the development of IBD and IBD-induced CRC. This review highlights how obesity-associated dysbiosis can lead to the pathogenesis of IBD and CRC with a special focus on mechanisms of altered absorption of short-chain fatty acids (SCFAs).

## 1. Introduction

Obesity is now a global pandemic, and its prevalence is increasing worldwide. According to the World Health Organization (WHO), in 2016, more than 1.9 billion adults (18 years or older) were overweight. Within this overweight category, 650 million adults were considered obese. In addition, in 2019, the incidence of childhood obesity was approximately 38.2 million. The prevalence of children and adolescents in the overweight and obese category has remarkably risen from 4% in 1975 to over 18% in 2016. Overall, the prevalence of worldwide obesity has increased three-fold since 1975 [1].

Obesity increases the risk for several chronic diseases including gastrointestinal (GI) disorders such as inflammatory bowel disease (IBD) and colorectal cancer (CRC) [2]. IBD is a condition that affects the digestive tract and is identified by chronic inflammation of the GI tract, affecting men, women, and children equally [3]. The pattern of inflammation varies between the two forms of IBD, namely, Crohn’s disease (CD) and Ulcerative colitis (UC). In UC, inflammation occurs in a continuous manner starting from the rectum and extending towards the colon, but UC is limited to the mucosa and submucosa with cryptitis and crypt abscesses. In CD, the inflammation affects all layers of the intestine (transmural inflammation) leading to strictures, fissuring ulceration, and granulomas [4,5,6]. People all over the world suffer from IBD, with the highest prevalence of IBD in industrialized countries such as North America and Europe. However, Eastern countries have also been observing a rise in IBD with increasing westernization [7]. Approximately 1.5 million Americans and 2.2 million Europeans suffer from IBD [8]. The incidence of pediatric IBD is about 100 or 200 per 100,000 children in the United States [9]. Although the cause of IBD is unknown, it is suggested that the complex interaction between genes, intestinal microbiota, inappropriate immune response, and environmental factors (lifestyle and diet) could influence IBD development and progression [10,11,12].

Moreover, IBD increases the risk for the development of CRC. The increased risk of CRC in IBD may be due to the chronic inflammatory state observed in IBD. Genetic alteration due to inflammation or mutations in TP53 (Tumor Protein 53) and KRAS (Kirsten rat sarcoma viral oncogene homolog) genes and gut dysbiosis is thought to be the cause of IBD-associated CRC development [13]. Around 10–15% of deaths that occur in IBD patients are due to colitis-associated CRC [14].

Overall, the pathology of both obesity and IBD present with disrupted intestinal homeostasis, including alterations in the gut microbiota. The gut microbiota shares a symbiotic relationship with the host, each benefiting from the other, making this an important factor to consider when studying the intestinal disease. The gut flora plays a significant role in the development of the host immune system, aids in breaking down and absorption of nutrients, provides protection from pathogenic infection, and maintains intestinal barrier function. In addition, microbes from the distal intestine (e.g., *Bacteroides*, *Bifidobacterium*, *Enterococcus*) also synthesize vitamins, mainly B12 and K. It has been shown that half of the daily requirement for vitamin K is provided by the gut bacteria [15]. Gut bacteria also produce short-chain fatty acids (SCFAs), which are significant mediators of intestinal homeostasis. The gut bacteria mainly mediate their actions through SCFAs. Over the past few decades, extensive research has shown that gut microbiota is linked to several diseases, and dysbiosis—i.e., an imbalance in the microbial composition and reduced diversity—is associated with the pathogenesis of various GI disorders such as IBD, obesity-mediated IBD, and IBD-mediated CRC [16].

## 2. Gut Microbiome

The gut microbiota is a complex community of bacteria, fungi, archaea, eukaryotes, and viruses that resides in the mammalian gut and has major influences on host health. The gut of a healthy individual harbors 100 trillion microbes with over 1000 bacterial species [17]. An adult human gut microbiome is predominated by the major phyla, including Firmicutes, Bacteroidetes, and minor quantities of Actinobacteria, Proteobacteria, and Verrucomicrobia [18,19]. Firmicutes are composed of Gram-positive bacteria with two main classes: Bacilli and Clostridia. Bacilli consist of obligate or facultative aerobes while Clostridia consists of only anaerobic microbes. Bacteroidetes, on the other hand, is composed of Gram-negative anaerobic bacteria. Actinobacteria (e.g., Bifidobacterium) contains Gram-positive anaerobic bacteria and Proteobacteria consists of (e.g., Escherichia, Klebsiella, Enterobacter) Gram-negative aerobic or facultative anaerobic bacteria [20]. The concentration of these bacteria varies along with the GI tract. The lowest concentration is observed in the stomach due to its acidic condition, while the highest concentration and diversity of bacteria is found in the large intestine due to higher pH. In addition to that, there is also a difference in the diversity and richness of bacteria between the lumen and the mucosa [21]. Most bacteria are found in the colon due to higher pH and longer transit time because most bacteria cannot survive in the low-pH environment of the stomach. The gut bacteria generate and release several metabolites such as short-chain fatty acids (SCFAs), bacteriocins, and antimicrobial peptides, which help to maintain GI homeostasis by providing energy and eliminating pathogenic bacterial overgrowth [22].

### 2.1. Early Colonization

Early colonization of bacteria in the gut occurs shortly after birth and is acquired mainly from the mother and some from the surrounding environment. Early colonization of bacteria in the human gut is affected by the mode of infant delivery (vaginal vs. cesarean), feeding process (breastfed vs. bottle-fed), and the type of bacteria present on the mother’s skin and mouth. As the body ages, colonization of bacteria depends on age, diet composition, geographical locations, medications, and stress [23]. By the age of three, the microbiome composition becomes stable and contains mainly the anaerobic species from the phyla Bacteroidetes, Firmicutes, and Clostridium cluster IV/XIV [22,24]. Early colonization by these beneficial bacteria is crucial as it allows the microbiota to educate the immune system by increasing tolerance to microbial determinants and preventing overgrowth of intestinal pathogens [25,26]. Besides, early colonization also influences gut microbiome composition throughout life. Research has shown that early colonization with the beneficial bacteria (e.g., Bacteroidetes, Firmicutes) is important in the prevention of childhood obesity [27,28,29].

### 2.2. Host Defense

The gut microbiotas share a mutualistic relationship with the host. The gut microbiotas provide nutrition, aid in mineral absorption, synthesize vitamins and amino acids, produce metabolites such as SCFAs, and protect the host against pathobionts (disease-causing bacteria). The gut bacteria possess a mechanism known as colonization resistance to ensure that the invading pathogenic bacterium senses the gut to be inhospitable. Colonization resistance can be of two types: microbe–microbe and microbe–host interactions.

Microbe–microbe interaction: Microbe–microbe interaction is important to prevent the growth of pathogenic bacteria in the gut and is a major part of colonization resistance.

Microbe–microbe interaction can lead to direct competition for space and nutrients limiting the pathogenic bacteria’s ability to establish and replicate within the lumen. By limiting access to nutrients, the indigenous bacteria suppress the growth of many pathogenic bacteria such as *C. difficile* [30].

In addition to competition for food or space, metabolic activities of the indigenous bacteria also inhibit the growth of invading pathogens. Metabolic activity produces SCFAs that can inhibit the expression of pathogenic bacteria such as *Enterobacteriaceae* [31]. Studies have shown that the SCFA butyrate downregulates the expression and decreases the ability of pathogenic *Salmonella enterica* to invade or induce apoptosis of host cells [31]. Bacterial production of SCFAs also reduces the pH of the lumen, which makes it difficult for pathogenic bacteria such as *Salmonella enterica* and *Escherichia coli* to survive. Metabolic activity of the indigenous bacteria also lowers oxygen concentration in the gut leading to the death of many pathogenic bacteria [32].

**Figure 1:**
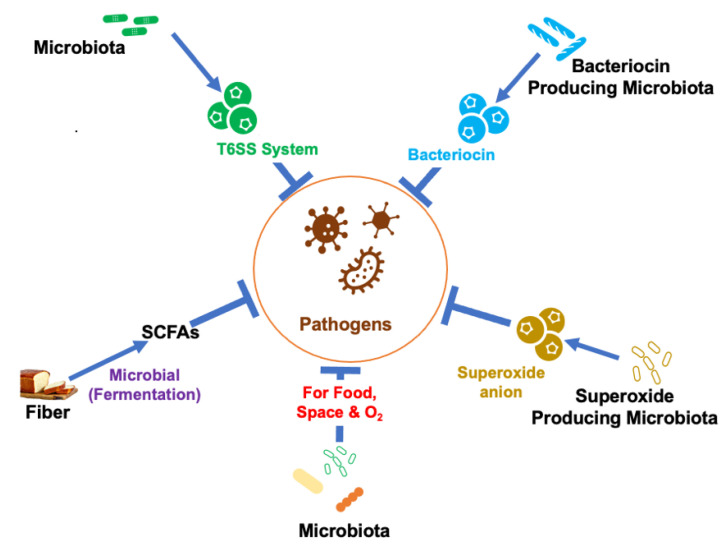
Mechanisms used by the gut bacteria in host defense. The gut microbiota modulates pathogenic bacterial overgrowth by producing antimicrobial peptides (bacteriocin), T6SS toxic protein, SCFAs, superoxide ion, and competing for food space and oxygen, which protects the host and maintains GI homeostasis. SCFAs: Short-chain fatty acids; O_2_: oxygen.

Some defensive bacteria such as *Enterococcus faecalis* also produce compounds such as superoxide anion that slows down the growth of many pathogenic bacteria such as *Staphylococcus aureus* [33]. Furthermore, the gut microbiota has a nanoweapon called the type VI secretion system (T6SS). T6SS is a protein secretion machine present in Gram-negative bacteria that secrets toxins directly in response to pathogenic bacteria. T6SS is important to control microbial community composition and overgrowth of pathogenic bacteria [34]. Additionally, the gut microbiota produces antimicrobial peptides (AMPs) such as bacteriocins to kill or inhibit pathogenic bacteria [35] (Figure 1).

Microbe–host interaction: Microbe–host interaction prevents inappropriate immune response and mucosal damage by the pathogenic bacteria. The host intestinal epithelial cells (IECs) of the mucosa provide a barrier function that separates the host cells from the gut flora, which avoids unfavorable interactions with host immune system. The host’s innate immune cells contain receptors known as pathogen recognition receptors (PRRs) such as Toll-like receptors (TLRs) and nucleotide-binding oligomerization domain-containing proteins (NODs) that recognize conserved microbial sequences, usually known as pathogen-associated molecular patterns (PAMPs). Until now, 10 TLRs in humans and 12 in mice have been discovered [36]. These receptors are expressed on the cell surface or in the endosomal membranes of IECs, hepatocytes, adipocytes, and immune cells such as macrophages and dendritic cells [37]. In the presence of lipopolysaccharides (LPS), peptidoglycans, nucleotides, proteins, and lipoproteins, these receptors are activated, triggering an immune response to protect the host. The production of AMPs by the host epithelial cells is known to be controlled by TLRs and NOD signaling. TLRs and NOD signaling are, in turn, regulated by gut microorganisms. A study showed that mice deficient in MyD88, a TLR signaling adaptor, showed reduced production of antimicrobial peptides such as RegIIIγ. Similarly, mice lacking the antibacterial peptide RegIIIγ had bacterial overgrowth in the intestine. RegIIIγ, produced from the ileum and mucus layer in the colon, is regulated by TLR stimulation and was shown to have a protective role against mucosal barrier function and prevention of translocation of pathogenic bacteria making the individual less susceptible to enteric infection and diseases such as IBD [38,39]. However, it is still not known how the TLR system distinguishes between commensal and pathogenic bacteria. The epithelial barrier function and integrity are further increased by the metabolites produced by the gut bacteria. SCFAs such as butyrate have been shown to increase mucus secretion by the goblet cells. Indole, a tryptophan metabolite, has also been shown to increase the expression of tight junction proteins, occludins, and claudins [37].

In another mechanism of microbe–host interactions, the host epithelial cells produce toxic compounds known as AMPs or host defense peptides. AMPs are secreted both by the gut microbiota and host epithelial cells. AMPs are shown to have broad-spectrum antimicrobial properties as they can kill both Gram-positive and Gram-negative bacteria [40]. AMPs are crucial for maintaining mucosal barrier function and preventing bacteria from reaching the epithelium. In the small intestine, epithelial barrier function is provided by AMPs secreted by the Paneth cells. In the large intestine, the mucosal barrier function is provided by the inner mucus layer composed of mucin 2, oligomeric mucus gel-forming (MUC2). The inner mucus layer also secretes AMPs. Finally, the gut microbiota protects the host barrier function by regulating the immune system. In rodent studies, segmented filamentous bacteria (SFB) has been shown to promote the differentiation of T helper 17 (Th17) and facilitate the production of interleukin (IL)-22 and immunoglobulin A (IgA). SFB are considered to be commensal bacteria that attach to the ileal epithelium and promote Th-17 differentiation. SFB also induces the production of IL-22 by the type III innate lymphoid cells (ILC3). The cytokines IL-17 and IL-22 upregulate RegIIIγ production by IECs, which helps to control both commensal and pathogenic bacteria overgrowth [37]. Together, the mutualistic relation between host and microbiotas help to prevent pathogenic bacterial overgrowth in the gut, maintain host integrity and barrier function, as well as develop the immune system, which are crucial to establish a healthy gut.

## 3. Short-Chain Fatty Acids (SCFAs)

### 3.1. Production of SCFAs

SCFAs are the major metabolites produced by bacterial fermentation of primarily dietary fibers. They are also produced in small amounts from proteins and peptides in the gut [41]. SCFAs are carboxylic acids with an aliphatic chain of 1–5 carbons including formic acid (C1), acetic acid (C2), propionic acid (C3), butyric acid (C4), and valeric acid (C5) (Table 1). The gut bacteria produce several types of SCFAs, but the major and most abundant SCFAs are acetate (C2), propionate (C3), and butyrate (C4), which are present in 60:20:20 proportion. The concentration of SCFAs varies along the length of the GI tract. The highest concentration of SCFAs is seen in the cecum and proximal colon followed by the distal colon, ileum, and jejunum. The concentration of SCFAs depends on the intake of dietary fiber and varies between individuals [42].

**Figure 2:**
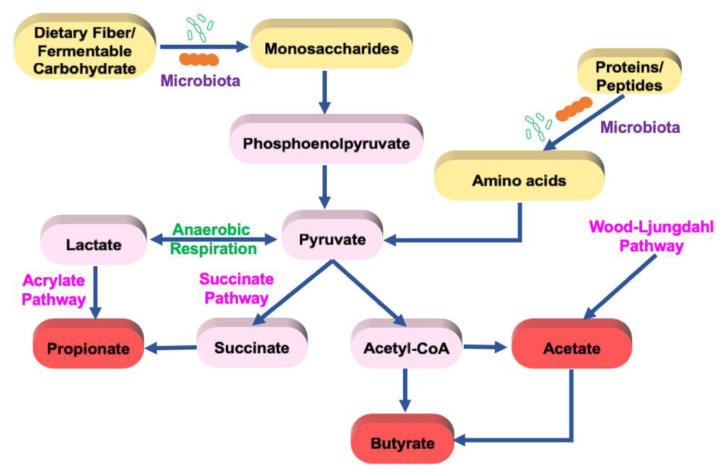
Pathways of SCFAs production by the gut microbiota. Gut bacteria produce SCFAs mainly by the following pathways: acetate is produced from acetyl-CoA (derived from the glycolytic pathway) or and Wood–Ljungdahl pathway, butyrate is produced from acetate and acetyl-CoA, and propionate is produced from the succinate and lactate through their respective pathways.

SCFAs are produced by the gut bacteria via multiple pathways (Figure 2). Acetate is produced from pyruvate via acetyl-CoA (derived from glycolysis) or the Wood–Ljungdahl pathway. Propionate is produced from succinate or lactate via the succinate and acrylate pathway, respectively. Butyrate is produced from acetyl-CoA and acetate. Butyrate and propionate are mainly produced from carbohydrate metabolism in glycolysis but can also be produced from organic acids and amino acid metabolism, whereas acetate is produced from acetyl-CoA [45]. The main SCFA-producing bacteria in the gut belong to the phyla Firmicutes, Actinobacteria, Bacterioidetes, and Verrucomicrobia [46,47,49,50,51].

### 3.2. Transport of SCFAs

Evidence shows that SCFAs play an important role in maintaining gut homeostasis. Therefore, understanding the transport mechanism of SCFAs is critical to maintaining host health. SCFAs produced by the gut bacteria in the lumen are absorbed by the IECs via two primary routes: (1) Nonionic (undissociated), by passive diffusion across the plasma membrane; (2) Anionic (dissociated), by carrier-mediated transport. Since the pKa of SCFAs is around 4.8 and the luminal pH is around 6.0, the majority of SCFAs are present in the dissociated form and transported into the IECs via carrier-mediated transport in their anionic form [52,53]. There are three main carrier-mediated transport mechanisms. (1) Monocarboxylic acid transporters (MCT1–4), (2) Sodium-coupled monocarboxylate transporter (SMCT/SLC5A), and (3) SCFA/HCO_3_^−^ exchangers. So far, 16 MCTs have been identified and, among these, MCT1–4 are SCFA transporters [54,55,56,57]. Table 2 shows the localization of MCTs (MCT1–4) among various species.

MCT1 (SLC16A1) is H^+^ coupled and the most studied MCT among the four. MCT1 cotransports SCFAs along with H^+^ in a H^+^: SCFA; 1:1 stoichiometric ratio. It is ubiquitously expressed throughout the GI tract but the expression of MCT1 is found to be highest in the cecum followed by the colon and low in the small intestine and stomach of humans, mice, and rats. The cellular localization of MCT1 is debated and not fully defined. Previous research showed that MCT1 is present either in the apical or basolateral membrane or both. According to Iwanaga et al., this variation could be due to different species or experimental conditions [62]. Besides transporting SCFAs, MCT1 can also transport lactate and pyruvate [53]. MCT1 was shown to be sensitive to α-cyano-4-hydroxycinnamate, bioactive flavonoids (e.g., quercetin and phloretin), thiol-modifying agents (e.g., p-chloromercuribenzene sulfonate), AR-C155858, and AZD3965 [52].

MCT2 and MCT3 follow similar transport mechanisms as those of MCT1. Although MCT2 and MCT3 are considered SCFAs transporters, there is only limited information present about them. MCT2 is expressed in the parietal cells of the hamster stomach but the localization of this transporter is not clear based on previous studies [62]. MCT3 is present at low levels in the basolateral membrane of the human ileum and colonic epithelial cells [60,62,63]. Kekuda et al. showed the presence of MCT4 in the apical membrane of rat intestinal cells (IEC-18 cell line). MCT4 is also present in myocytes for lactic acid transportation and in mouse small intestinal villus and crypt cells. In humans, MCT4 is present on the basolateral membrane of IECs [62,64]. Although the SCFA butyrate was shown to be transported by both MCT1 and MCT4, MCT4 has a higher affinity for butyrate. It has been shown that knockdown of MCT4 by small interfering RNA (siRNA) exhibits 40% inhibition of butyrate transport in the IEC-18 cell line [64].

SMCTs belong to the SLC family of solute carriers. So far, two members from the SLC5 gene family have been characterized as sodium-coupled monocarboxylate transporters, namely, SMCT1-2. SMCTs that are very similar to MCTs in substrate specificity. SMCT1(SLC5A8) was initially discovered as a tumor suppressor. It is a Na-dependent SCFA transporter that facilitates SCFAs transport in the presence of sodium in a Na^+^: SCFA; 2:1 stoichiometric ratio and is localized at the apical membrane. The expression of SMCT1 varies along the GI tract with the higher expression in the lower GI tract compared with the upper GI tract. Although SMCT1 is a Na-dependent transporter, Cui et al. showed that this transporter could be inhibited by substrates of MCTs (e.g., lactate, pyruvate, and butyrate), nonsteroidal anti-inflammatory drugs (e.g., ibuprofen, ketoprofen, and naproxen), and probenecid [65]. SMCT2 (SLC5A12) is a low-affinity SCFAs transporter, unlike SMCT1 which is a high-affinity SCFAs transporter. SMCT2 is also localized at the apical membrane similar to SMCT1, but the expression of SMCT2 is limited to the small intestine with no detectable expression in the colon [52]. SMCT2 co-transports SCFA and sodium in a Na^+^: SCFA; 1:1 stoichiometric ratio.

SCFA/HCO_3_^−^ exchangers are low-affinity and high-capacity SCFA exchangers, though the molecular and genetic identity of these transporter is still unknown. SCFA/HCO_3_^−^ exchangers are present on both the apical and basolateral membranes and are shown to carry out different functions. The apical SCFA/HCO_3_^−^ exchangers are responsible for the influx of SCFAs from the intestinal lumen into the cell while the basolateral SCFA/HCO_3_ are responsible for the efflux of SCFAs from the cell into systemic circulation. The affinity (1/*K_m_*) of the apical SCFA/HCO_3_^−^ exchangers is much higher than the basolateral SCFA/HCO_3_^−^ exchangers (*K_m_* = 1.5 mM vs. 17.5 mM) [53,66]. Several studies showed that SCFAs uptake via SCFA/HCO_3_^−^ exchangers is bicarbonate- and pH-dependent and is greatly enhanced by low luminal pH. Few studies reported SCFA/HCO_3_^−^ exchangers to be sensitive to 4,4′-diisothiocyanatostilbene-2,2′-disulfonic acid (DIDS) [64,67], while others showed them to be insensitive to DIDS [68].

**Figure 3:**
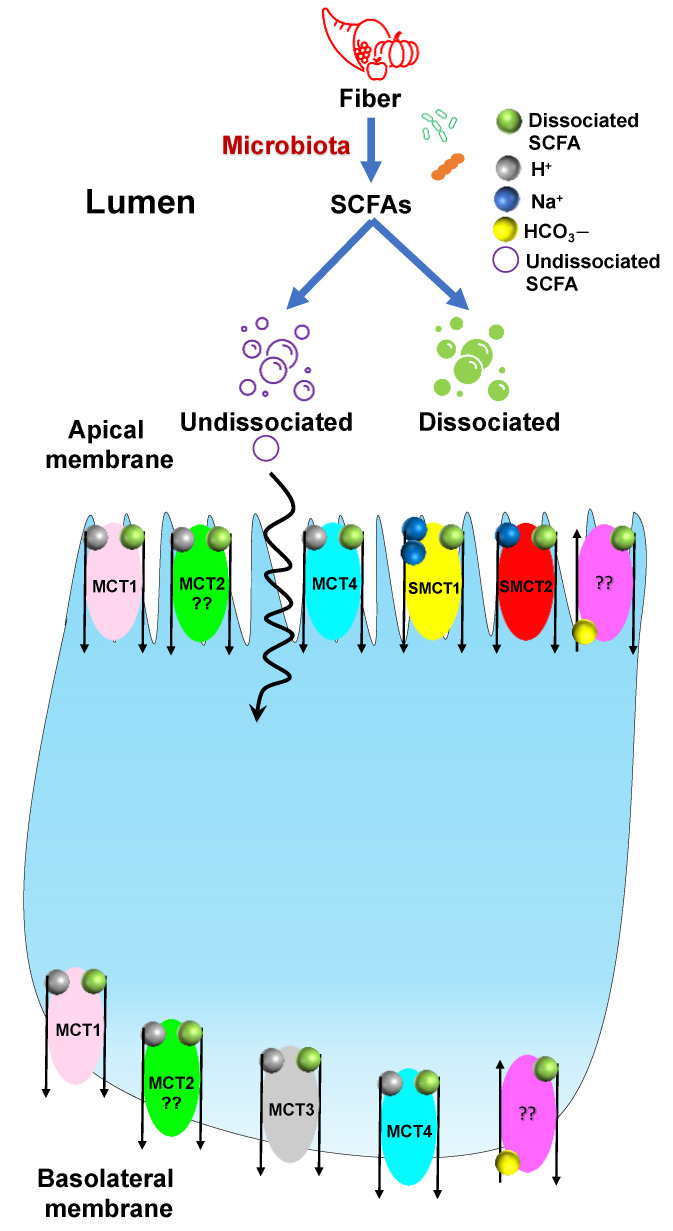
An overview of SCFA transport mechanisms. SCFAs produced in the gut lumen by the gut flora exist in two forms: undissociated and dissociated SCFAs. Undissociated SCFAs can pass across the apical plasma membrane by passive diffusion while the dissociated SCFAs require the aid of transporters. MCT1–4, Monocarboxylic acid transporters (shown in light-pink, green, grey, and blue); SMCT1–2, Sodium-coupled monocarboxylate transporter (shown in yellow and red); SCFA/HCO_3_^−^ exchangers (shown in magenta).

Taken together, MCT1, SMCT1, and SCFA/HCO_3_^−^ exchangers are the SCFAs transporters. Thus, SCFAs are important to maintain intestinal homeostasis and their transport into the intestinal epithelium is critical to prevent chronic intestinal diseases. Figure 3 shows an overview of SCFAs transport mechanisms.

### 3.3. SCFAs as Activators of Signaling Pathways

In addition to the intracellular functions of SCFAs that require transport into the intestinal epithelium, SCFAs also elicit beneficial effects on IECs via extracellular mechanisms. Extracellular mechanisms involve interactions with the G-protein-coupled receptors (GPCRs). GPCRs are the largest group of membrane receptors in mammals and carry out a myriad of physiological functions in the body. The three main SCFAs—acetate-, propionate-, and butyrate-regulated GPCRs—are GPR41 (aka free fatty acid receptor (FFAR)-3), GPR43 (aka FFAR2), and GPR109A (aka hydroxycarboxylic acid receptor (HCAR)-2). These receptors are expressed in the enteroendocrine L cells of the intestine, mast cells, and leukocytes. SCFA-activated GPCRs are responsible for controlling intestinal inflammation, immune cell development, and epithelial barrier function [67,68,69,70]. A study showed that mice deficient in GPR41(^−/−^) and GPR43(^−/−^) had delayed immune response upon 2,4,6-trinitrobenzenesulfonic acid (TNBS)-induced colitis compared with control mice. GPR43 activation by SCFAs has been shown to regulate neutrophil chemotaxis, recruitment of inflammatory mediators, development of regulatory T (T_reg_) cells, and activation of the inflammasome pathway in colonic epithelium, which is essential in maintaining intestinal integrity and homeostasis. GPR41 and GPR43 regulate tissue inflammation by activating the downstream mitogen-activated protein kinase (MAPK) pathway [69,70]. Studies showed that GPR41 and GPR43 play an important role in regulating glucose and lipid metabolism by modulating the secretion of glucagon-like peptide (GLP)-1, peptide YY (PYY), insulin, and leptin hormones [71,72]. GPR109A stimulates the differentiation of colonic T_reg_ cells and controls inflammation by suppressing the expression of nuclear factor kappa light chain enhancer of activated B cells (NF-κB). GPR41 and GPR43 can be activated by acetate, propionate, butyrate, and other SCFAs, whereas GPR109A can be activated by butyrate and niacin [73].

## 4. Effect of SCFAs on Health

SCFAs exert numerous beneficial effects on host health. SCFAs play an important role in the regulation of host energy metabolism, chloride absorption, inflammation, gut barrier function, development of the immune system, and maintaining oxidative status [74,75]. Some of the known health benefits of SCFAs are briefly discussed below.

### 4.1. SCFAs and Gut Barrier Function

**Figure 4:**
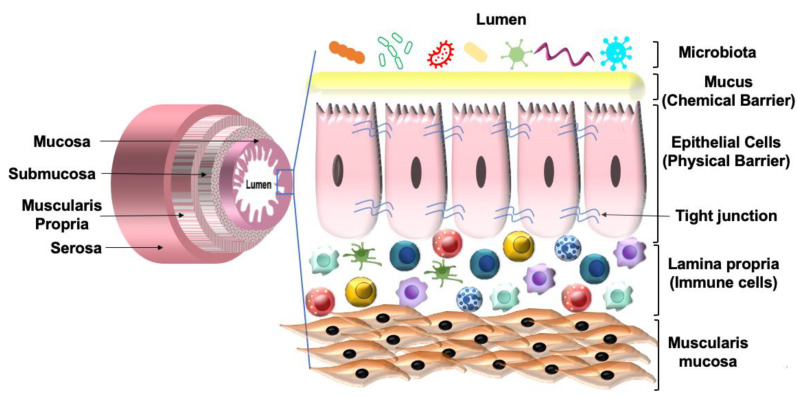
Schematic representation of the various layers of the GI tract. Mucus acts as a chemical barrier and separates the epithelial cells from the luminal contents while the epithelium and tight junction proteins provide a physical barrier to maintain gut integrity.

The GI tract consists of four layers of specialized tissues that include the mucosa, submucosa, the muscularis propria, and the serosa. The mucosa is the innermost layer and surrounds the lumen of the GI tract. The mucosa is made up of three layers: the epithelium; the lamina propria, which is made up of thin layers of connective tissue; and the muscularis mucosae consisting of thin smooth muscle. The lamina propria is present below the epithelium and contains both innate and adaptive immune cells. The epithelium provides a physical barrier that is facilitated by tight junction proteins. The interaction of the tight junction proteins with the cytoskeleton forms a complex structure that limits gut permeability and paracellular movement. Besides the physical barrier, the intestinal epithelium also provides a chemical barrier facilitated by the mucus layer secreted by the goblet cells of the epithelium. The mucus layer separates the epithelium from the luminal contents, which prevents an inappropriate immune response. Together, the physical and chemical barriers of the intestinal epithelium help maintain gut integrity and homeostasis (Figure 4). Any alteration to barrier function can lead to chronic intestinal diseases such as IBD, celiac disease, irritable bowel syndrome (IBS) and CRC. Factors that are associated with altered barrier function include overgrowth of pathogenic bacteria such as enteropathogenic *E. coli*, high-fat diet (HFD), LPS, nonsteroidal anti-inflammatory drugs (NSAIDs), proton pump inhibitors (PPIs), food allergens, and gluten component gliadin [76,77].

SCFAs play a vital role in regulating the chemical and physical barrier functions. Studies have shown that SCFAs are positively correlated with gut barrier functions. Luminal administration of butyrate and acetate was shown to increase MUC2 production by goblet cells in the rat colon [53]. In one study, mucositis-induced mice showed improved intestinal permeability and reduced ulceration upon oral butyrate supplementation [78]. Another investigation showed that SCFAs, mainly butyrate, enhanced tight junction protein expression and reduced intestinal permeability in a LPS-induced porcine intestinal epithelial cell line (IPEC-J2) [79]. Butyrate is thought to mediate its effect by activating AMP-activated protein kinase (AMPK) and stabilizing hypoxia-inducible factor (HIF). Mice fed with fermentable dietary fiber showed increased production of SCFAs and increased expression of tight junction proteins (zona occludens (ZO)-1, ZO-2, occludin, junctional adhesion molecule A (JAMA), and claudin-7). Another study showed that diet-induced obese C57BL/6 mice (*ob/ob*) fed on a prebiotic diet with SCFAs showed reduced gut permeability and increased tight junction proteins (ZO-1 and occludin) [51]. In a dextran sodium sulfate (DSS) BALB/c mouse model of colitis, administration of guar gum (fiber) for 12 days increased fecal SCFAs compared to controls and showed a 60–120% increase in tight junction proteins, mainly occludin and claudins-3, -4, and -7 [80]. Moreover, dietary supplementation of sodium butyrate was seen to improve gut barrier function and expression of host defense peptides in the intestine and restore gut homeostasis in deoxynivalenol (DON)-induced intestinal epithelial dysfunction in piglets [53]. Collectively, SCFAs play a major role in the maintenance of gut barrier function.

### 4.2. SCFAs and Anti-Inflammation

**Figure 5:**
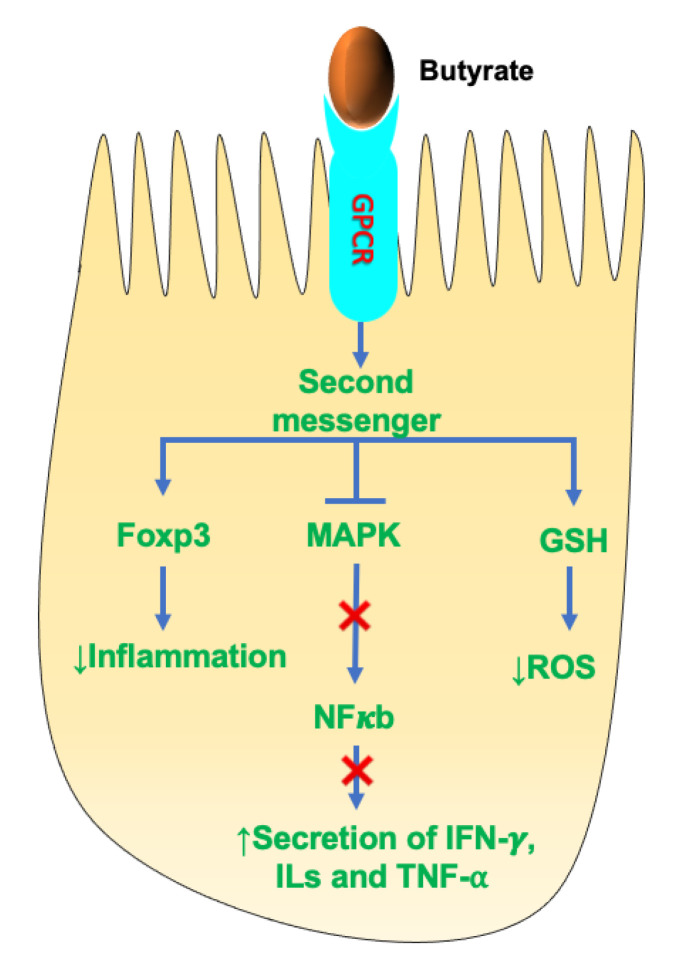
Schematic representation of the mechanism of SCFA butyrate on immune modulation. Butyrate binds to G-protein-coupled receptors (GPCRs), which activate various downstream signaling pathways involved in the regulation of inflammation and ROS production. SCFA, short-chain fatty acid; Foxp3, forkhead box protein P3; MAPK, mitogen-activated protein kinases; GSH, glutathione; NF-κB, nuclear factor κB; IL, interleukin; (IFN)-γ, interferon; TNF-α, tumor necrosis factor; ROS, reactive oxygen species. Upward arrow denotes increase and downward arrow denotes decrease.

SCFAs are strong modulators of the host immune system. SCFAs regulate T-regulatory (T_reg_) cell differentiation, immune cell chemotaxis, expression of proinflammatory cytokines and production of reactive oxygen species (ROS). Figure 5 shows the mechanism of SCFAs in immune modulation in IECs.

SCFAs, primarily butyrate and propionate, exhibit anti-inflammatory properties. Studies have shown that butyrate regulates the expression of the anti-inflammatory forkhead box protein P3 (Foxp3), which is crucial in reducing the inflammatory response [81]. In addition, butyrate was also shown to modulate the secretion of several proinflammatory mediators (e.g., interferon (IFN)-γ; IL-1, 2, 6, 8; tumor necrosis factor (TNF)-α). These effects of butyrate were mainly due to suppression of NF-κB and MAPK pathways. Suppression of NF-κB was shown by all three major SCFAs, but butyrate was the most effective and acetate the least effective [82]. Another study showed that SCFAs (butyrate) alleviated the effect of ROS by stimulating the expression of the antioxidant glutathione (GSH). Much of the anti-inflammatory and immunomodulatory effects of SCFAs are mediated by binding to GPCRs. Butyrate was shown to upregulate IL-10 expression and downregulate IL-6 expression by binding to GPCR109A present on dendritic cells (DC) and macrophages. Few clinical studies on humans showed that butyrate administration in UC patients showed reduced intestinal inflammation and improved colitis symptoms [83,84]. Moreover, acetate was shown to modulate intestinal inflammation by interacting with the GPCR43 receptor. Studies showed that GPRC43^−/−^ knockout in germ-free mice exacerbated intestinal inflammation [81]. Although acetate is not a powerful anti-inflammatory agent, studies showed that both acetate and propionate decreased the release of LPS-induced TNFα in human neutrophil culture media [81].

The principal mechanisms of SCFAs inhibiting inflammation in the gut are by inhibiting histone deacetylase (HDACs), activating histone acetylase (HATs) and stabilizing HIFs. HDACs have been shown to regulate gene expression, where overexpression reduced gene transcription leading to gene silencing. Moreover, HDAC inhibitors are extensively used in cancer therapy due to their anti-inflammatory and immunosuppressive properties. HDAC inhibition by SCFAs was shown to increase *Foxp3* expression in T_reg_ cells. Together, SCFA-mediated HDAC inhibition produces immunological tolerance, immune homeostasis, and an anti-inflammatory cellular phenotype [53,81,82,85].

### 4.3. SCFAs and Energy Metabolism

A substantial amount of SCFAs, particularly butyrate, are utilized by the colonocytes for energy following production by the gut bacteria. Butyrate provides around 60–70% of energy requirements for colonocyte proliferation and differentiation [86]. A study showed that germ-free mice lacking butyrate showed reduced expression of enzymes involved in fatty acid metabolism in the mitochondria, which resulted in decreased oxidative phosphorylation and adenosine triphosphate (ATP) production leading to autophagy. These effects were attenuated by butyrate administration [87]. Following utilization by the colonocytes, the remaining SCFAs reach the systemic circulation through the portal vein and are used as a substrate for several metabolic processes. Approximately 10% of the daily caloric requirement of the host is provided by SCFAs [88].

Research showed that SCFAs regulate appetite and body weight. Acetate, the most abundant SCFA produced by gut bacteria, is quickly absorbed in the proximal colon and transported to the liver, where it is used as a substrate for cholesterol synthesis. Acetate modulates appetite and body weight by directly stimulating the anorectic pathways in the hypothalamus and brainstem. Acetate administration in HFD-fed mice showed a significant reduction in food intake. Moreover, acetate administration increased expression of pro-opiomelanocortin (POMC) and decreased expression of agouti-related peptide (AgRP) causing a change in appetite [89].

About 80% of the propionate produced by the gut flora is taken up by the liver, where it is used as a substrate for gluconeogenesis, lipogenesis, and protein synthesis [41,45] (Table 3).

In addition to acetate, propionate and butyrate were also seen to regulate body weight. A study showed that 10 g/day of propionate supplementation along with the regular diet in human subjects for 24 weeks prevented weight gain compared with controls. Several studies have also showed a correlation between butyrate administration and weight loss. Goa et al. showed that butyrate incorporation in HFD for 16 weeks showed a significant decrease in the body weight of HFD-induced C57BL/6J male obese mice [53]. Further, Li et al. reported that integration of butyrate to HFD for 9 weeks reduced diet-induced obesity by 27% in mice fed with HFD supplemented with butyrate compared with controls fed only HFD [90]. Furthermore, Arnoldussen et al. showed that butyrate supplementation was effective for long-term weight management (mice over 10 months old) in obese mice fed with HFD for 12 months [91]. Apart from these studies, other studies showed that supplementation of a mixture of the three SCFAs (acetate, butyrate, propionate) suppressed HFD-induced weight gain [92,93].

SCFAs regulate glucose metabolism by activating GPCRs. Studies have shown that butyrate and propionate increase PYY secretion by the L-cells in the ileum and colon by stimulating the GPCR41 receptor. PYY slows down gastric emptying, increases digestion and absorption of nutrients (glucose), and reduces food intake by promoting satiety [94,95]. Further, the SCFAs (butyrate, acetate, and propionate) regulate the secretion of GLP-1 by stimulating GPCR43 receptors. GLP-1 controls blood glucose levels by regulating the secretion of insulin and glucagon [96]. A study by Sakakibara et al. showed that, in addition to insulin and glucagon, SCFAs also regulate leptin secretion by activating GPCR43 receptors [97].

SCFAs were also shown to regulate lipid metabolism and energy expenditure. Acute oral administration of acetic acid was shown to increase energy expenditure and lipid oxidation in C57BL/6J mice [98]. Another investigation reported that HFD supplemented with a mixture of the three SCFAs (acetate, propionate, and butyrate) increased energy expenditure and lipid oxidation in C57BL/6J mice [99,100]. In addition, Besten et al. showed that butyrate supplementation suppresses peroxisome proliferator-activated receptor gamma (PPAR-γ) expression and activity and increases lipid oxidation [100]. Clinical studies in humans showed that acute oral sodium propionate supplementation in healthy male and female volunteers enhanced resting energy expenditure and lipid oxidation compared with controls [99]. Similarly, another study showed that rectal infusion of the physiological concentration of SCFAs present in the colon (200 mM) stimulated lipid oxidation, reduced carbohydrate oxidation, and increased resting energy expenditure [99]. Taken together, SCFAs play a crucial role in regulating food intake and energy metabolism.

## 5. Diet–Microbiome Interaction

### 5.1. Effect of Diet on Microbiota

Diet is an important modulator of human health and is linked to several pathophysiologies such as obesity, IBD, and CRC. Through diet, we receive all the macro- and micronutrients that are beneficial for the human body to function. A healthy diet is not only advantageous to human health but also to the microbes that reside in the gut as they acquire the energy and nutrients from the host’s diet. A study by Tierney et al. showed that there are 22 million microbial genes in the gut and 24 million in the mouth, forming a total of 46 million bacterial genes in the human body [101]. This huge population of the microbiome is crucial to maintaining host physiology as the number of microbial genes outnumbers genes in the human genome. These microbes mainly feed on nondigestible carbohydrates (complex carbohydrates and fibers) that the host cannot break down. The microbiotas possess the necessary enzymes to degrade these complex sugars and use them as their key source of energy [102,103].

Western diet profoundly alters gut composition. A diet rich in saturated and trans fats causes gut dysbiosis. In Sprague–Dawley (SD) rats, a Western diet composed of high fat and high sucrose reshaped gut microbiome composition and caused metabolic dysfunction within 3 days [104]. Moreover, rats fed on a HFD showed less *Lactobacillus intestinalis* and more *Clostridiales*, *Bacteroides*, and *Enterobacteriales*. *Lactobacillus intestinalis* were shown to be negatively correlated with the rat’s fat mass and body weight [105]. On the other hand, research showed that a diet composed of complex carbohydrates is associated with the production of beneficial microbiota *Bifidobacterium* and *Lactobacillus*. Members of these species are associated with improved barrier function, production of SCFAs, modulation of inflammation, lipid metabolism, and increased absorption of minerals [106,107,108,109]. In addition, complex carbohydrates have been seen to increase the abundance of *Ruminococcus*, *E. rectale*, and *Roseburia,* which are the major butyrate-producing bacteria. Reduced production of butyrate has been associated with CRC and IBD. A clinical study with 344 patients with advanced colorectal adenoma showed reduced butyrate production due to decreased numbers of butyrate-producing bacteria—*Clostridium*, *Roseburia*, and *Eubacterium* spp.—while pathogenic bacteria *Enterococcus* and *Streptococcus* spp. were increased compared with healthy controls. The reduction of butyrate production was due to low fiber intake in the advanced colorectal adenoma group, suggesting a positive correlation with diet and colorectal adenoma [110]. In IBD, a low fecal count of *Roseburia* was reported. The reduction in *Roseburia* and *Eubacterium rectale* was associated with a high-protein diet [111,112]. Furthermore, when an animal-based diet (meats, eggs, and cheeses) was compared with a plant-based diet (grains, legumes, fruits, and vegetables) using 10 subjects (six males, four females) for 5 consecutive days, altered microbiome composition was observed. The animal-based diet caused a reduction in Firmicutes levels and increased the population of bile-tolerant microbes (such as *Alistipes*, *Bilophila*, and *Bacteroides*) while the plant-based diet had an increased abundance of species from the Firmicutes phyla (*Roseburia*, *Eubacterium rectale*, and *Ruminococcus bromii*) [113].

### 5.2. Effect of Diet on SCFAs

SCFAs (acetate, propionate, and butyrate) are crucial in maintaining intestinal homeostasis. Almost 95% of SCFAs result from microbial fermentation and present in a 60:20:20 proportion (acetate: propionate: butyrate) in the colon and feces. However, the total concentration of SCFAs truly depends on the type of diet consumed by the host [114,115]. A diet with little or no complex carbohydrates is shown to reduce SCFAs production [102]. Mice fed a conventional diet containing 3.3% crude fiber had increased SCFAs production compared with the mice that received a synthetic diet with no fiber. This difference in SCFAs production was due to the alterations in microbiome composition. The synthetic-diet group showed reduced SCFAs producing bacteria in the small intestine [116]. Another study showed mice that received dietary supplementation of cycloinulooligosaccharides had higher SCFAs in the small intestine [117]. Mueller et al. showed the effect of macronutrients on circulating SCFAs concentration. The study was conducted with 164 subjects in a crossover trial who were given three different diets (carbohydrate, plant protein, and fat) enriched with high fiber (~30 g/2100 kcal) for 6 weeks. The results from the study showed that acetate was the predominant SCFA in circulation and the protein–fiber diet produced more acetate than the carbohydrate–fiber diet. Propionate was decreased in carbohydrate and fat–fiber diet and butyrate were increased with protein–fiber diet [118].

Since the gut microbiome relies solely on the host for food, consuming a healthy diet is paramount. A healthy diet rich in indigestible carbohydrates is ideal for a healthy microbiome composition as it increases the richness and diversity of beneficial microorganisms [119]. In addition, fermentation of indigestible carbohydrates and proteins by the gut bacteria produces metabolites such as SCFAs that exert beneficial effects on host health.

## 6. Obesity

Obesity is a complex disease that is associated with numerous adverse health outcomes including heart disease, Type 2 Diabetes (T2DM), high blood pressure, and certain cancers, primarily due to the accumulation of excess fat in the body. Several factors (e.g., genes, hormones) contribute to obesity, but the most important are environmental factors (sedentary lifestyle, diet). Numerous studies have shown that the overconsumption of a Western diet (high sugar, high fat) is the leading cause of obesity and obesity-associated metabolic disorders [120,121]. Consumption of a Western diet in combination with a sedentary lifestyle causes an imbalance between caloric intake and energy expenditure leading to a positive energy balance [122]. Prolonged positive energy conditions cause adipocyte hypertrophy (increase in cell size), which leads to hypoxia and stress, which further triggers inflammatory response resulting in adipocyte dysfunction. Dysfunctional adipocytes release more free fatty acids (FFA) causing ectopic accumulation of fat around the liver, heart, intra-abdominal region (visceral tissues), skeletal muscle, and pancreas due to impaired adipogenesis and increased lipolysis. In addition to that, adipocyte dysfunction also leads to the secretion of several proinflammatory adipokines causing low-grade inflammation, which is associated with metabolic disorders (obesity, T2DM, diabetes, dyslipidemia, and hypertension) [123,124]. Extensive research has shown that the Western diet also causes gut dysbiosis, connecting obesity and the gut microbiota [125].

### 6.1. Obesity and Gut Microbiota

Obesity is associated with changes in gut microbiota composition. The gut microbiota of obese patients is less diverse with a reduced relative abundance of gut bacteria compared to normal [126,127]. In a study, fecal analysis in genetically obese mice (*ob/ob*) showed a 50% decrease in Bacteroidetes and increased Firmicutes compared with the lean mice. Similarly, fecal microbiota analysis in 12 obese humans also showed an increased Firmicutes/Bacteroidetes ratio [126]. However, this decrease in Bacteroidetes was shown to improve with weight loss in obese individuals [126]. Besides Firmicutes and Bacteroidetes phyla, the gut of obese individuals also showed a reduced proportion of Verrucomicrobia and an increased proportion of Actinobacteria [128]. Although an overall increase in Firmicutes was observed in obese humans and mice there was a decrease in the *Faecalibacterium prausnitzii* species from the Firmicutes phylum [129,130,131]. *Faecalibacterium prausnitzii* is associated with the reduction in low-grade inflammation in obesity and diabetes [132]. *Faecalibacterium prausnitzii* is a commensal bacterium with anti-inflammatory properties. It is a dominant species in healthy adults and the main butyrate-producing bacterium in the intestine. There are contradictory results found in literature about *Faecalibacterium prausnitzii* in obesity. Some groups have shown it to increase [133] in obesity while others have shown it to decrease [130]. However, the reason for this conflicting finding is not clear. Even though adipocyte dysfunction could be a major contributor of obesity, multiple studies have shown that alteration of the gut microbiota may result in obesity [134,135]. Turnbaugh et al. found that the gut microbiota of obese mice has the capability to harvest more energy from food compared with nonobese microbiota, and transplantation of obese microbiota with germ-free mice resulted in significantly higher body weight in the germ-free mice [136]. This change suggests that obese animals harbor a unique gut microbiome composition that may be associated to a HFD. Despite several studies, it is still unclear whether obesity is a cause or consequence of gut dysbiosis; thus, more studies are required to clarify this contradiction. In the context of obesity and gut microbiota, the specific roles of LPS and SCFAs in obesity is further discussed below.

#### 6.1.1. Role of LPS

Obesity is in part characterized by low-grade inflammation and studies have showed that low-grade inflammation can be caused due to the host’s innate immune response to LPS. LPS are also known as endotoxins and are present in the outer membrane of Gram-negative bacteria. LPS activates the immune system by binding to their receptor complex (CD14/TLR4/MD2), promoting the secretion of proinflammatory cytokines resulting in chronic low-grade inflammation. TLR4 activation by LPS was shown to increase adiposity by the upregulation of inflammatory pathways such as c-Jun N-terminal kinase and NF-κB in adipocytes and macrophages [137].

**Figure 6:**
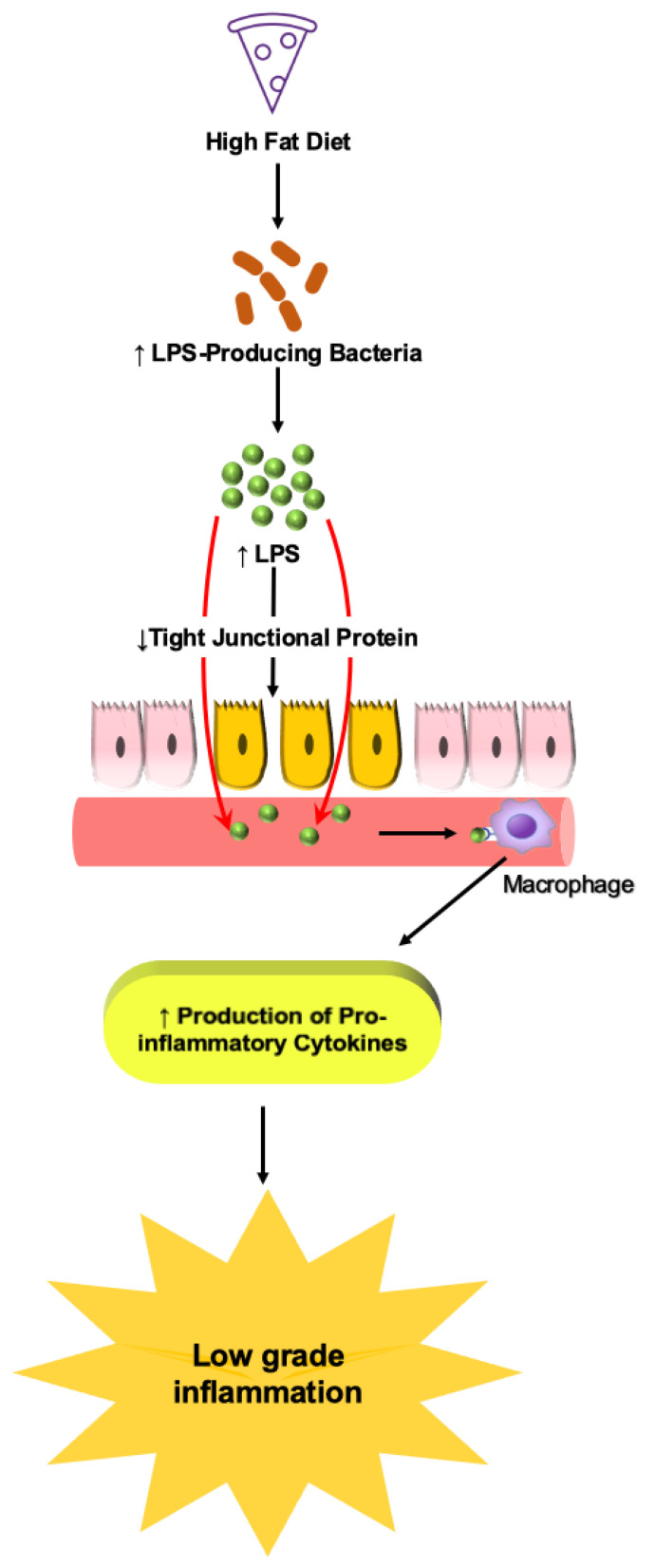
Mechanism of LPS-mediated immune response. High-fat diet increases the population of LPS producing bacteria in the gut. LPS producing bacteria downregulates the expression of tight junction proteins, which in turn increases intestinal permeability and translocation of LPS. In the circulation, LPS binds to its receptor on immune cells and increases the release of proinflammatory cytokines causing low-grade inflammation. LPS, lipopolysaccharide. Upward arrow indicates increased; downward arrow indicates downregulation.

A healthy individual usually has low levels of LPS in circulation. However, research has shown that obese individuals or experimental models of diet-induced obesity are associated with high levels of LPS in circulation causing endotoxemia. Cani et al. showed that B6 mice fed a HFD (72% fat) for 4 weeks exhibited a high plasma concentration of LPS, suggesting that a HFD favors the growth of more LPS-producing Gram-negative bacteria [138]. Gram-negative bacteria reduce the expression of tight junction proteins such as ZO-1 and occludin, increasing intestinal permeability and, thus, causing metabolic endotoxemia (Figure 6) [139]. Cani et al. also showed that subcutaneous LPS infusion in mice for 4 weeks resulted in fasted glycemia, insulinemia, and weight gain. In addition, LPS infusion also caused increased adipose tissue F4/80-positive cells (a marker for macrophage), inflammatory markers, and liver triglyceride content. All these effects of LPS infusion were similar to those seen in HFD-fed mice [138].

#### 6.1.2. Role of SCFAs

SCFAs produced by gut bacteria may also play a significant role in obesity. Butyrate and propionate are considered anti-obesogenic due to their ability to improve metabolic syndromes (T2DM, obesity) [140,141], while acetate is considered obesogenic. SCFAs are used as a substrate in several physiological processes. SCFAs, mostly acetate, are used as substrates for lipogenesis and cholesterol synthesis in the liver and other tissues. SCFAs upregulate carbohydrate-responsive element-binding protein (ChREBP) and the sterol regulatory element-binding transcription factor 1 (SREBP1) involved in lipogenesis. In addition, SCFAs can repress the fasting-induced adipocyte factor (FIAF), which inhibits lipoprotein lipase (LPL), inducing accumulation of triglycerides (TG) in host adipocytes, because LPL breaks down TG to be used by the body for energy [142]. SCFAs also decrease fatty acid oxidation by suppressing adenosine monophosphate kinase (AMPK), which results in increased accumulation of fat [143]. Some microbes are better at extracting energy from food than others. Research has shown that the microbes from the Firmicutes phylum increase absorption of calories resulting in weight gain and obese animals and human subjects tend to have higher levels of Firmicutes [144]. Furthermore, microbes from the Firmicutes phylum increase fatty acid absorption and decrease fatty acid oxidation, contributing to more fat accumulation and weight gain [145].

### 6.2. Obesity and IBD

#### 6.2.1. Epidemiology and Risk Factor

IBD patients, particularly CD, are traditionally presented with low body mass index (BMI) and body weight as they suffer from severe diarrhea and are considered malnourished [146,147]. However, recent studies showed the prevalence of obesity and overweightness in IBD patients in both adult and pediatric cases. [148,149]. A study conducted by Subihne et al. demonstrated that 40% of CD patients were overweight/obese compared with 52% of controls. In addition, a higher BMI was shown to be positively correlated with higher C-reactive protein (CRP) in those patients. Although further studies are required to investigate the relation between high BMI and CRP in IBD, CRP is a marker for inflammation and has been implicated in IBD and obesity [150]. Another study by Steed et al. showed that 18% of CD patients were obese and 38% overweight with only 3% of patients being underweight. The authors of this study concluded by confirming the prevalence of obesity and overweightness in the general population [151]. Few other studies reported the prevalence of obesity in IBD patients. A cohort study of 316,799 children from Copenhagen School Health demonstrated a direct association between childhood BMI and CD and concluded by confirming obesity as a risk factor for CD [152]. In contrast with the above studies, the European Prospective Investigation into Cancer and Nutrition Study (EPIC) found no association between obesity and the development of CD or UC [153]. This variation in association between obesity and IBD could be due to age and gender, since the former two studies were conducted among children or young adults.

A retrospective cohort study, the Swiss Inflammatory Bowel Disease Cohort Study (SIBDCS), examined the impact of obesity on disease activity and disease course in patients with IBD. They found that 11% of IBD patients were obese and disease activity scores in the obese CD patients were elevated, but not in UC patients. They also showed that obesity decreased rates of disease remission and increased the course of disease complication with no effects on disease progression [154]. In another study, Uko et al. investigated the significance of obesity as a risk factor in CD. They found that CD patients had higher visceral adipose tissue volume (VAT) compared with healthy controls at diagnosis. Increased VAT is associated with systemic inflammation. The authors concluded that higher VAT volume in CD patients is associated with higher disease activity at diagnosis, increased complications, and hospitalization [155].

#### 6.2.2. Impact of Obesity on IBD

In the literature, the role of obesity on IBD, mainly CD, is inconsistent and controversial [156]. Some groups showed that obesity measured by BMI had a severe disease outcome. Obese individuals with IBD had frequent perineal complications and hospitalizations, requiring the need for early surgery, presented with extraintestinal manifestations, and showed poor surgical outcome when compared with nonobese IBD patients [157,158,159]. In addition, Matthew et al. showed that obese IBD children undergoing intestinal resection had a 2-fold higher risk for hospital readmission compared with average BMI patients [160]. Similarly, obesity measured by visceral adiposity also showed severe disease outcome. Erhayiem et al. found that high mesenteric fat index (MFI), defined as the area of ratios of visceral-to-subcutaneous fat, was a marker of aggressive CD. Others also reported that VAT/subcutaneous ratio was a better indicator of disease association and activity compared with BMI [161,162].

On the contrary, other investigators have found no plausible link between obesity and IBD, mainly CD. Seminerio et al. found that though high BMI severely impacted the patient’s quality of life, no additional hospitalizations or surgeries were necessary. Another group showed that obese IBD individuals required less hospitalizations or surgeries compared with normal/underweight patients. Pringle et al. showed that the obese CD patients had low risk of strictures and perianal complications [163,164,165]. Similarly, Kim et al. found no significant differences between obese IBD with either CD or UC with nonobese IBD patients [166]. These conflicting findings, though unclear, could be due to the methods used to measure obesity. Further research, both basic and clinical, is indeed required to clearly define a plausible link between obesity and IBD.

### 6.3. Obesity in the Pathogenesis of IBD

Obesity is characterized by an increase in total body fat. Anatomically, fat is stored mainly in two main compartments, subcutaneous and visceral. VAT includes intra-abdominal adipose tissue, comprising mesenteric, omental, and retroperitoneal adipose tissue. In obesity, expansion of all adipose depots takes place. Visceral adiposity is considered a risk factor for metabolic syndromes and many inflammatory diseases including GI diseases [167,168,169].

A retrospective study conducted by Rowan et al. demonstrated increased visceral adiposity in CD patients but not in UC [169]. Magro et al. showed increased visceral fat in CD patients compared with controls. In this study, visceral fat was measured using dual-energy X-ray absorptiometry (DEXA) scans [170]. In a retrospective study by Buning et al., patients with CD had significantly higher visceral fat, which was associated with stricturing/fistulizing, and higher disease activity compared with patients with no stricturing/fistulizing [171]. Several other groups have reported the association of visceral adiposity being a risk factor for higher disease activity in CD [161,172].

In comparison with the above studies, Yadav et al. found no association between visceral adiposity and disease activity in both CD and UC. This difference could be due to patient population as this study only included Indian patients and the pathomechanism of IBD might be different for Asian patients [173].

#### 6.3.1. Role of Adipose Tissue

Adipose tissue is an endocrine organ that is composed of adipocytes, macrophages, lymphocytes, fibroblasts, progenitor cells, and endothelial cells and is responsible for the secretion of leptin, adiponectin, cytokines, vascular regulators angiotensin II, and plasminogen activator inhibitor (PAI) [174]. Obesity is associated with adipocyte dysfunction resulting in the alteration of normal physiology of adipocytes leading to increased production of proinflammatory cytokines and decreased production of adiponectin, increased synthesis of CRP, and increased lipolysis, which results in the activation of the signaling pathways such as inhibitor of nuclear factor kappa-B kinase subunit beta (IKK-β) and NF-κβ leading to low-grade inflammation. Activation of these transcription factors is found to be reported in obese subjects [175,176].

Numerous clinical studies have shown an association between obesity and IBD, mainly CD, as the pathologies of these diseases share similar features, including adipocyte dysfunction, gut dysbiosis, and inflammation [177,178]. Clinical studies have shown that obese individuals have a higher risk of developing IBD [178,179] due to higher visceral fat volumes [180].

Most studies found in the literature associated visceral adiposity to obesity, and very few studies looked at the association between obesity and mesenteric dysfunction. A study conducted by Yang et al. showed that mesenteric adipose tissue (MAT) collected from obese diabetic subjects had high gene expression of leptin, PPAR-γ, fatty acid translocase (FAT/CD36), and 11β hydroxysteroid hydrogenase (HSD) suggesting that alteration in the mesenteric depot may play a role in the development of metabolic syndrome [181]. Others reported that the term VAT is used interchangeably with MAT as visceral adiposity is the accumulation of fat in the intra-abdominal region [169]. Therefore, in obesity, an increase in visceral fat may also mean an increase in mesenteric fat. However, future studies on abdominal adiposity and its link with obesity should focus on looking at all the compartments of fat depots in the abdomen and not just visceral fat.

**Figure 7:**
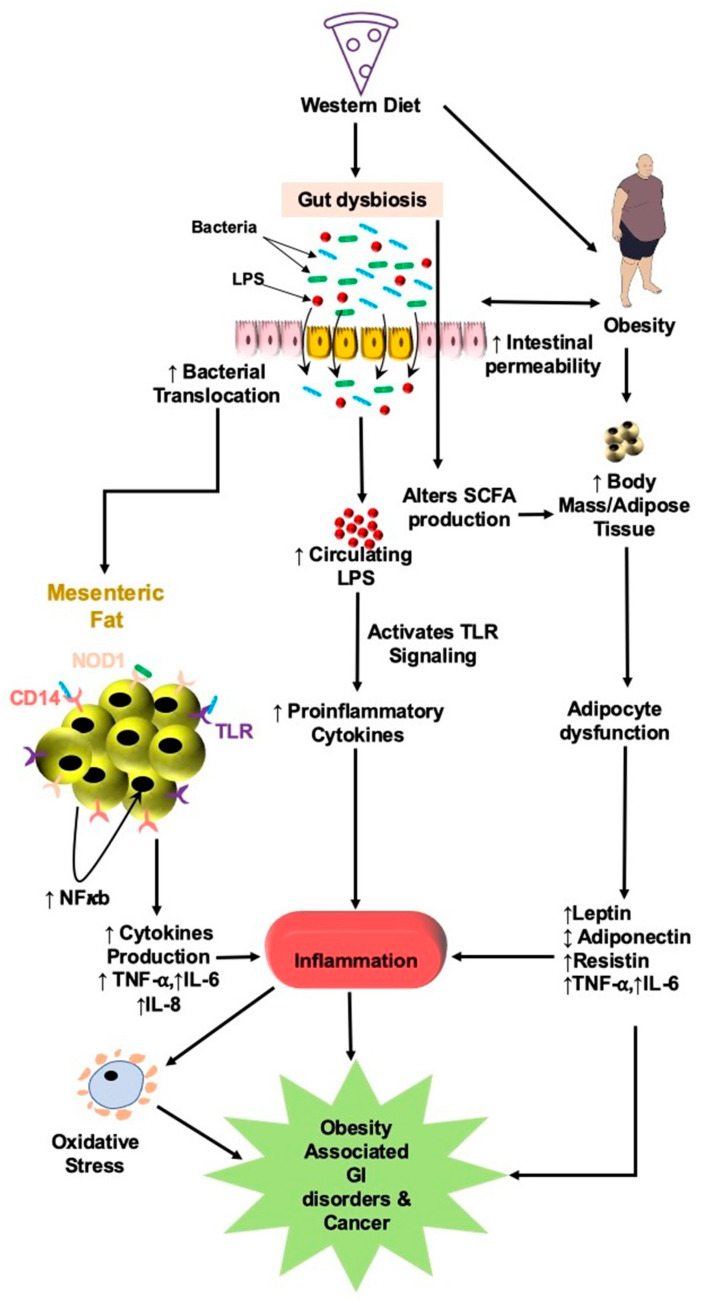
Pathomechanism of GI disorders (CD, UC) and cancer in obesity. The interplay between gut microbiota, adipose tissue, immune system, and intestinal permeability leads to the development of GI disorders and cancer. Dysbiosis increases intestinal permeability and alters SCFAs production. Defective intestinal barrier translocates bacteria into the mesenteric adipose tissue resulting in the production of proinflammatory cytokines. Defective barrier also increases circulating LPS causing inflammation. Adipocyte dysfunction alters adipokine secretion which can participate in low-grade inflammation. Thus, the complex interaction between diet, obesity and dysbiosis creates an inflammatory milieu which predisposes to GI disorders and cancer. LPS, liposaccharide; TLR, toll-like receptor; CD14, cluster of differentiation 14; NOD1, nucleotide binding oligomerization domain containing 1; NF-kB, nuclear factor kappa B; IL-6,8, interleukin; TNF-α, tumor necrosis factor alpha; SCFA, short-chain fatty acid. Upward arrow represents increase; downward arrow represents decrease; double head arrow indicates either increase or decrease.

In obese individuals, excess fat deposition leads to adipocyte hypertrophy and secretion of proinflammatory cytokines (TNF-α, and IL-6), chemokines, and complement factors causing low-grade inflammation. Accumulating studies showed a link between altered mesenteric fat (aka creeping fat) and IBD, mainly in CD [182,183]. Low-grade inflammation, on the other hand, can cause an imbalance between leptin/adiponectin ratio and increase intestinal permeability, bacterial translocation, and T-cell infiltration, thus, predisposing an obese individual to IBD [184], as depicted in Figure 7. Karmiris et al. showed that downregulation of leptin expression in mesenteric fat may be due to the inflammatory milieu in IBD patients due to increased production of TNF-α [185]. However, other conflicting studies have shown that leptin levels increased or remained unchanged in IBD [186,187]. More research is required to fully define the role of leptin expression in IBD.

Obesity is also associated with low adiponectin levels. Low levels of adiponectin are observed in IBD. In an in vivo study conducted on 120 female Kunming mice, investigators showed that adiponectin reduced DSS-induced rectal bleeding in mice. Further, adiponectin decreased the expression of proinflammatory cytokines (IL-1β and TNF-α) and increased the expression of tight junction proteins (ZO-1 and occludin) in DSS-induced mice. In addition to this in vivo study, an in vitro study showed that adiponectin improved barrier function and reduced inflammatory mediator expression in Caco-2 cells [188]. On the contrary, the role of adiponectin is debated in IBD. One study has in fact reported overexpression of adiponectin in IBD [189].

Resistin is an adipokine that has been found to be elevated in obesity. Resistin has been shown to increase expression of IL-6, IL-12, TNF-α, and monocyte chemotactic protein 1 (MCP-1). In a study conducted by Astrid et al., high serum resistin levels in both CD and UC patients were demonstrated. The authors found that resistin concentration was associated with disease activity in IBD and concluded that resistin may be an independent predictor of disease activity in IBD, mainly CD [190]. Overall, the inflammatory milieu associated with obesity may play a central role in the development of IBD.

#### 6.3.2. Role of Gut Microbiome

Diet-induced obesity is associated with alteration of the gut microbiota. A Western diet rich in high-fat, high-sugar foods reduces the number of butyrate-producing bacteria (e.g., *Clostridiales*, *Eubacterium rectale*, *Roseburia intestinalis*) and increases the number of opportunistic bacteria (e.g., *Bacteroides caccae*, *Escherichia coli*). Obesity also reduces the number of *Faecalibacterium prausnitzii*, which is the main butyrate-producing bacteria and possesses anti-inflammatory properties [191]. In addition, HFD-induced obesity increases the number of Proteobacteria and decreases the number of Bacteroidetes [192]. A study by Leung et al. showed that increased body weight is positively correlated with higher *Clostridium difficile* infection in IBD [193]. Quantitative polymerase chain reaction (qPCR) analysis on colonic biopsy samples from IBD patients showed a marked reduction of *F. prausnitzii* in adult IBD patients and a decrease in *A. muciniphila* in pediatric IBD patients (below 16 years of age) [194]. Another study showed that mice fed with a high-fat/high-sugar diet had gut dysbiosis, increased intestinal permeability, increased mucin-degrading bacterium *Ruminococcus torques*, and increased colonization of adherent-invasive *Escherichia coli* (AIEC) bacteria in the gut mucosa leading to inflammation, which was also observed in IBD patients (CD) [195]. Furthermore, a Western diet is also associated with the alteration of SCFAs production and absorption in IBD. Mice fed with HFD for 18 weeks showed reduced expression of the SCFA receptor (GPCR43) on ileal mucosa [196]. In UC and CD, expression of the SCFA transporter MCT1 was reduced, and butyrate uptake and oxidation were inhibited in UC. The change in SCFA transporter and receptor expression may be due to inflammation or alteration of gut microbiome composition [197,198]. Overall, alteration in the gut microbiota due to a HFD causes a reduction in SCFAs-producing bacteria—mainly butyrate-producing bacteria—and an increase in pathogenic bacteria, affecting the production and absorption of SCFAs. Overgrowth of pathobionts disrupts barrier function and causes inflammation, which results in the downregulation of SCFAs’ receptor and transporter, as observed in IBD patients and in in vivo models of IBD.

### 6.4. Creeping Fat (CF)

In IBD, mainly CD, adipose tissue is considered a risk factor in the pathogenesis of the disease. MAT is the fat around the mesentery and attaches the intestine to the abdominal wall. In CD, MAT hyperplasia often takes place and is shown to correlate with transmural inflammation, fibrosis, and stricture formation. Altered MAT is referred to as creeping fat that wraps around the inflamed intestine and is a pathologic characteristic of CD but not UC. Creeping fat (CF) is considered a more biologically active fat compartment and may be the primary source of proinflammatory cytokines (IL-β, IL-6, TNF-α) responsible for inflammatory processes in IBD. It has been shown that the expression of cytokine correlates with adipocyte mass [199]. In addition, mesenteric hyperplasia positively correlates with increased serum CRP. CRP production in CD patients is a marker for inflammation. Elevated CRP levels may be due to the interaction between adipocyte and bacterial antigens as high leptin and TNF-α levels in the MAT may induce chronic inflammation in the adjacent intestine, which in turn may damage the intestinal wall translocating bacteria into the MAT since mesenteric fat is in proximity with the intestine and expresses TLR and NOD1 receptors.

Recently, few laboratories reported the presence of microbiota in CF. Ha et al. investigated the role of microbial translocation in the development and expansion of CF. In this study, the authors found the presence of viable *Clostridium innocuum* in the MAT collected from surgical resections. Further, single-cell RNA sequencing identified CF as profibrotic and proadipogenic enriched with activated immune cells responding to microbial stimuli. This was confirmed by using gnotobiotic mice colonized with *Clostridium innocuum.* Ex-vivo data demonstrated that *Clostridium innocuum* stimulates M2 macrophage remodeling leading to an adipose tissue barrier that prevents spreading of bacteria in circulation. The author concluded that development of CF might be a protective response to restrict bacterial translocation into the circulation that may have been migrated from damaged intestine. However, the authors also mentioned that this response has no off-switch and it may be the fat contributing to the severe intestinal scarring observed in 40% of the CD patients [200].

Another study by Amar et al. exhibited that HFD-induced diabetes increases intestinal permeability and translocates mucosal adherence bacteria of *Enterobacteriaceae* into the MAT causing low-grade bacteremia. These bacteria then colocalize with the dendritic cells in the lamina propria. They also showed that this mechanism requires CD14 and NOD1 receptors expressed on MAT (Figure 7). The authors concluded that this mechanism could be considered as a therapeutic strategy to control HFD-induced diabetics and metabolic syndrome [201].

He et al. showed that presence of MAT microbiota was associated with the development of CD and translocation of *Achromobacter pulmonis* into the MAT exacerbates colitis symptoms [202]. Gummesson et al. showed that visceral adiposity measured by waist circumference is positively correlated with increased permeability [203]. Similarly, Massier et al. reported the presence of microbiota in adipose tissue of obese or T2D subjects. The authors found presence of bacterial DNA (mainly from Firmicutes and Proteobacteria phyla) in the human omental, subcutaneous, and mesenteric adipose tissues. Mesenteric fat was the primary site of translocated bacteria, and the quantity was correlated with inflammation [204].

In comparison, Zulian et al. found no association with microbiota and human adipose tissue [205]. This variation could be due to sampling issues. Bacterial translocation occurs also in healthy gut. In CD, the translocation of bacteria increases due to increased intestinal permeability. This results in increased cytokine production and infiltration of immune cells promoting inflammation and disease severity.

## 7. IBD and CRC

Although the incidence of CRC is about 1–2% among the general population, approximately 15% of deaths that occur in IBD patients are due to CRC [206]. IBD, particularly UC, increases the risk of CRC. The chronic inflammatory state of IBD is the main cause of CRC and the survival rate is low. The most important risk factor in IBD-associated CRC is the duration and extent of IBD. Several groups have shown that patients with long-standing UC and CD have higher risk for developing CRC compared with general population [207].

### 7.1. Inflammation

Inflammation is a well-known risk factor for GI disorders and cancer. Inflammation in the GI tract may cause continuous turnover of cells in the lining of the intestine that may increase the chance of irregularities leading to cancer formation. Chronic inflammation is evident in 20% of human cancers and the long-standing inflammation is manifested in CRC, which was confirmed by pharmacological suppression of inflammation [208]. IBD is characterized as chronic inflammatory disorder. Several groups have reported the upregulation of inflammatory markers in IBD. Calprotectin, a neutrophil-derived protein, has become an established marker for whole gut inflammation. In IBD subjects, levels of this protein were associated with bowel inflammation [209,210]. Increased cytokine production (IL-6, IL-1, TNF-α) observed during IBD is known to influence cancer initiation and progression as cytokines can promote growth and prevent apoptosis while also facilitating invasion and metastasis. Circulating IL-6 levels are shown to positively correlate with CRP levels and CRP was shown to be elevated in obesity and IBD. Activation of TNF-α was shown to activate NF-κB, which was shown to be upregulated in 50% of CRC [211,212]. Inflammation also leads to the production of reactive oxygen species (ROS) and nitrogen species causing oxidative stress, which has been associated with carcinogenesis [213,214]. Increased expression of nitric oxide synthase (NOS), oxygen, and nitrogen species were found in the inflamed tissues of patients with CD or UC [215,216].

### 7.2. Gut Microbiota

Intestinal microbiotas have also been associated with a major role in CRC. According to one study conducted using 24 male SD rats of 6 weeks of age, which were divided into two groups and had UC and CRC induced, a shift in the gut microbiome was observed between the control group and the UC and the CRC group. In control vs. UC and CRC induced SD rats, a shift from Firmicutes, Verrucomicrobia, and Actinobacteria to Proteobacteria, Firmicutes, and Verrucomicrobia was observed. In the UC group, higher levels of *Enterobacteriales*, *Enterobacteriaceae*, *Escherichia–Shigella*, and species from Proteobacteria were observed compared with controls with even higher levels in the CRC group [217]. Similarly, UC and CRC group showed fewer Firmicutes species—*Bacilli*, *Lactobacillaceae*, and *Lactobacillus*—which were much lower in CRC. *Lactobacillus* from the Firmicutes phylum was also shown to have anti-inflammatory properties [217].

Altered gut microbiota produces metabolites that can also promote the development of UC to CRC. Studies revealed that *Enterobacteriaceae* (*Enterobacteriales*) and Proteobacteria are involved in the metabolism of arachidonic acids and linoleic acids, the levels of which were found to be higher in UC and CRC induced SD rats [217]. Metabolism of these inflammatory mediators has been linked to the development of CRC [218]. In addition, *Enterobacteriaceae* has also been reported to promote cancer [219]. Altered gut microbiota composition also favors the growth of pathogenic bacteria contributing to inflammation by activation of TLR, toxin secretion and invasion, or adherence. *E. coli* was shown to contribute to inflammation by activating TLR4 and facilitating the development from UC to CRC [220,221]. *E. coli* also activates NF-κB expression, which plays a significant role in inflammation and CRC development [222]. Taken together, alterations in the gut microbiota produce metabolites that facilitate the development and progression of CRC in IBD.

## 8. Obesity and CRC

Obesity has been considered a risk factor for CRC. Dai et al., in a meta-analysis study, showed that obesity measured by BMI increases the risk for CRC in men but not in women. However, when obesity was measured by waist-to-hip ratio (WHR) the relationship between obesity and CRC was consistent for both men and women. Further analyses showed that risk of cancer was high in obese individuals measured by WHR and waist circumference than BMI. The authors concluded by considering obesity as a statistically significant risk factor for CRC [223]. Another study by Claudia et al. showed that obesity worsens the condition of azoxymethane/dextran sodium sulfate (AOM/DSS) induced colitis-associated carcinoma (CAC) in mouse. The authors demonstrated that diet-induced obesity impairs gut barrier function resulting in increased inflammation and immune cell recruitment promoting CAC. The proposed mechanism displayed that obesity-associated IL-6-induced macrophage polarization recruits lymphocytes via the chemokine ligand/chemokine receptor (CCL-20/CCR 6) axis accelerating CAC formation [224]. Liu et al. also showed obesity to be a risk factor for early onset of CRC among women. In this study, the authors found that higher BMI at early age (18 years old) and weight gain since early adulthood were associated with the increased risk of early onset CRC, suggesting that obesity and weight gain since early adulthood accelerates CRC development at an early age [225].

### Mechanisms

Obesity is associated with altered adipokine secretion. Low adiponectin and high leptin levels are observed in obesity. A study showed that this alteration could favor CRC development as adiponectin is a negative regulator of angiogenesis and could inhibit CRC growth. In vitro and in vivo studies found leptin to be an antiapoptotic, proangiogenic, and proinflammatory. Research also found a positive correlation between circulating leptin concentration and CRC growth. Fatty acid synthase overexpression is observed in obesity and has been shown to be associated with the CRC phenotype. Resistin is another adipokine that has been shown to be associated with CRC. A meta-analysis study by Yang et al. showed that higher circulating resistin levels are associated with increased risk for colorectal cancer [226,227].

Gut dysbiosis may also play a role in CRC. A study by Campisciano et al. showed that the microbiota profile of obese and CRC subjects is similar, suggesting a role of obese microbiota in tumor formation. A higher abundance of *Proteobacteria* and *Verrucomicrobia* was found in CRC subjects, which was also observed in obese subjects. Within these two phyla, the authors found the presence of two bacteria, *Hafnia alvei* (*Proteobacteria*) and *Akkermansia muciniphila,* in tumor and obese groups. These are mucin-degrading bacteria and overexpression of mucins MUC1 and MUC5AC seen in CRC patients may be a consequence [228]. Dysbiosis also affects the microbial signature, which can lead to inflammation and tissue damage.

## 9. Conclusions

Western diet and lifestyle are associated with adverse health outcomes. Western diet causes obesity and gut dysbiosis. Obesity is recognized as a significant risk factor for several disorders, including GI disorders. Whether altered gut microbiota is a cause or consequence of obesity remains unclear, but dysbiosis is observed in obesity as well as in GI disorders. The involvement of obesity in IBD patients is becoming more common and has been shown to play a role in the development and course of the disease. Several mechanisms have been postulated in this regard. The role of adipose tissue has been demonstrated in the pathomechanism of IBD. Altered adipocyte function and deregulated production of adipokines, such as leptin, adiponectin, resistin, and proinflammatory cytokines (IL-6, TNF-α), leads to chronic inflammation that may predispose an obese individual to IBD, mainly CD. In CD, mesenteric fat dysfunction (creeping fat) also secretes altered adipokine and cytokines as seen in obesity. Although mesenteric fat dysfunction in obesity is controversial, few groups have reported the role of mesenteric fat in metabolic syndrome. High leptin and TNF-α production from mesenteric fat may induce chronic inflammation in the adjacent intestine, causing damage to the intestinal wall and translocating bacteria to the mesenteric fat causing it to creep to the small bowel. Bacterial translocation can also occur due to increased intestinal permeability due to consumption of a western diet. The presence of this fat causes scarring and fibrosis, a common feature of CD. The role of gut microbiota has also been implicated in IBD. Altered gut microbiota and SCFA composition have been observed in IBD patients. Dysbiosis causes an imbalance in the equilibrium between proinflammatory and anti-inflammatory bacteria and metabolites disrupting intestinal homeostasis propagating the pathogenesis of the disease. The long-standing inflammation in obesity and IBD is a predisposing factor of CRC. Inflammation may cause DNA damage by the production of reactive oxygen and nitrogen species causing oxidative stress and increasing cell turnover number leading to cancer formation.

This review summarized the role of gut microbiota and their metabolites in the maintenance of health as well as in the pathogenesis of diseases. The review discussed key types of gut microbiota and their metabolites such as SCFAs, which are altered in obesity and are associated with chronic diseases such as IBD and CRC. This review also discussed how the gut microbiota is possibly involved in the onset and progression of these diseases. Recently published studies have identified intracellular signaling pathways that are known to regulate gut dysbiosis-mediated alterations in gut function. However, it is important to decipher key receptor-mediated downstream signaling pathways in the intestinal epithelial cells to identify novel therapeutic targets for precise therapeutic modulation in chronic diseases of the gut.

## Figures and Tables

**Table 1:** Major and minor short-chain fatty acids (SCFAs) produced by the gut microbiota.

	Name	Lipid No.	Major Producers	References
	Acetate	C2:0	*Lactobacillus* spp. *, *Bifidobacterium* spp., *Akkermansia muciniphila*, *Bacteroides* spp., *Prevotella* spp., *Ruminococcus* spp., *Streptococcus* spp.	[43,44]
Major SCFAs	Propionate	C3:0	*Phascolarctobacterium succinatutens*, *Akkermansia muciniphila*, *Bacteroides* spp., *Dialister* spp.,*Megasphaera elsdenii*, *Veillonella* spp., *Coprococcus catus*, *Roseburia inulinivorans*, *Ruminococcus obeum*, *Salmonella* spp.	[45,46]
	Butyrate	C4:0	*Faecalibacterium prausnitzii*, *Clostridium leptum*, *Eubacterium rectale*, *Roseburia* spp.	[47]
Minor SCFAs	Formate	C1:0	*Bifidobacterium* spp., *Prevotella* spp., *Parabacteroides* spp., *Bacteroides* spp., *Alistipes* spp., *Eubacterium* spp., *Erysipelatoclostridium* spp., *Blautia* (*Clostridium cluster XIVa*) spp., *Coprococcus*, *Dorea*, *Roseburia* (*Clostridium cluster XIVa*) spp., *Lactobacillus* spp., *Faecalibacterium* (*Clostridium cluster IV*) spp., *Ruminococcus* (*Clostridium cluster IV*) spp., *Streptococcus* spp., *Veillonella* spp., *Escherichia* spp.	[48]
	Valerate	C5:0	*Clostridium* (*Clostridium cluster I*) spp.	[48]

* spp.: species.

**Table 2:** Distribution and localization of monocarboxylic acid transporters (MCTs) among various species.

Transporter	Species	Cell/Tissue-Type	Localization	References
MCT1	Hamster	IECs	Basolateral membrane	[58]
	Human, Rat, Pig	IECs	Apical membrane	[59,60,61]
	Human, Mouse, Rat	Enterocytes	Basolateral membrane	[62]
MCT2	Hamster	Parietal Cells	Unknown	[62]
MCT3	Human	Ileum, Colon	Basolateral membrane	[60,62,63]
MCT4	Rat	IEC-cell line	Apical membrane	[62,64]
	Mouse	IECs (Villus and Crypts)	Basolateral membrane	[62,64]
	Human	Ileum, Colon	Basolateral membrane	[62,64]

**Table 3:** Sites of short-chain fatty acids (SCFAs) absorption and their functional role in energy metabolism.

SCFAs	Absorption	Site of Utilization	Function	References
Butyrate	Colonocytes	Colon	Differentiation and proliferation of colonocytes	[86]
Acetate	Proximal Colon	Liver	Regulation of appetite, body weight, and cholesterol synthesis	[89]
Propionate	Colonocytes	Liver	Used for gluconeogenesis, lipogenesis, and protein synthesis	[41,45]

## Data Availability

Not applicable.
